# Supplementary figures and images for: Open-source data management system for Parkinson’s disease follow-up
Source: PeerJ Comput Sci. 2021 Feb 17;7:e396. doi: 10.7717/peerj-cs.396 (PMC7959639; doi:10.7717/peerj-cs.396)

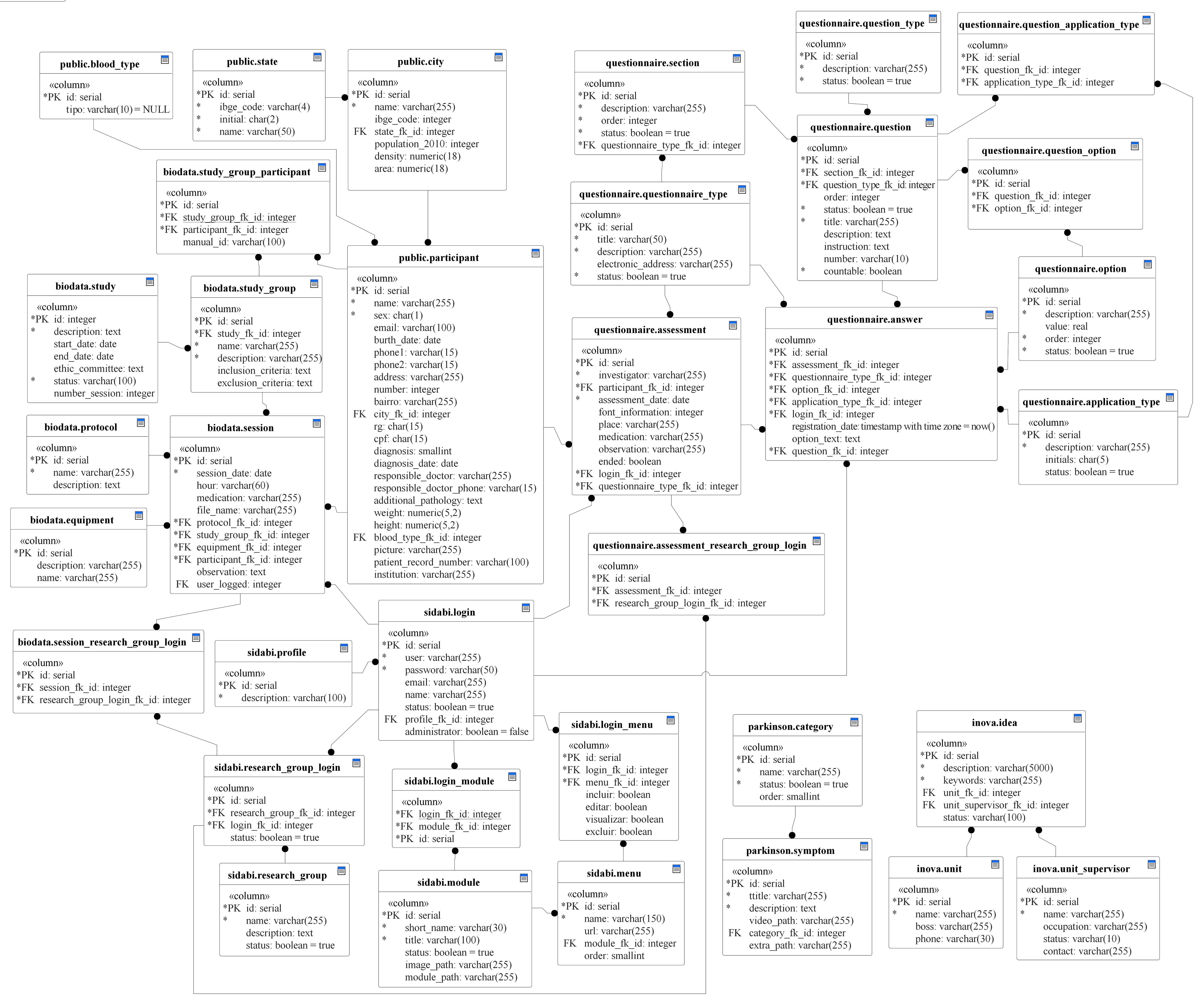

Supplement: Supplemental Information 1 [file peerj-cs-07-396-s001.png]
